# Comparison of 25 µg Vaginal Misoprostol Versus Dinoprostone Gel for Induction of Labor in Term Prelabor Rupture of Membranes: A Retrospective Record-Based Observational Study

**DOI:** 10.7759/cureus.116739

**Published:** 2026-09-22

**Authors:** Shweta Jinger, Priyanka Songara, Kshatrapal Dabi

**Affiliations:** 1 Department of Obstetrics and Gynecology, Government Satellite Hospital Chopasni Housing Board (CHB), Dr. S.N. Medical College, Jodhpur, IND; 2 Department of Obstetrics and Gynecology, Charbhuja Hospital, Merta, IND; 3 Department of General Surgery, Mathuradas Mathur (MDM) Hospital, Jodhpur, IND

**Keywords:** bishop score, cervical ripening, dinoprostone gel, induction of labor, prelabor rupture of membranes, vaginal misoprostol

## Abstract

Background and objective: Prelabor rupture of membranes (PROM) at term occurs in 8-10% of pregnancies, and a long latency period is associated with infection in both the mother and the newborn. In women with an unfavorable cervix, cervical ripening and induction of labor are widely used with prostaglandin analogs. However, the preferred agent in women with term PROM remains unclear. This study compared the efficacy and safety of 25 µg vaginal misoprostol (prostaglandin E1) and dinoprostone (prostaglandin E2) gel in women with term PROM. Materials and methods: This was a record-based, comparative, observational study with a retrospective design conducted at a tertiary care teaching hospital in West India over 12 months. Records of 200 women with term PROM (≥37 weeks), singleton cephalic pregnancy, and an unfavorable cervix (Bishop's score ≤6) were reviewed. The primary outcome was the induction-to-delivery interval and the proportion of vaginal deliveries within 24 hours. Secondary outcomes included change in Bishop score at six hours, oxytocin dose, interval from induction to active labor, need for oxytocin augmentation, mode of delivery, and maternal and neonatal safety outcomes. An independent-samples t-test was employed for continuous variables, and chi-square or Fisher's exact test was used for categorical variables; p < 0.05 was considered significant.

Results: The two groups did not differ in maternal age, parity, PROM duration, or baseline Bishop score. The mean Bishop score at six hours was significantly higher in the misoprostol group (5.68 ± 1.31 vs 4.78 ± 1.26; p < 0.001). The mean induction-to-active-labor interval (6.80 ± 1.58 vs 8.19 ± 2.06 hours; p < 0.001) and the induction-to-delivery interval (11.81 ± 3.06 vs 13.78 ± 3.99 hours; p < 0.001) were significantly shorter with misoprostol, which also required fewer doses (1.62 ± 0.63 vs 1.91 ± 0.67; p = 0.002) and less oxytocin augmentation (16 (16.0%) vs 46 (46.0%); p < 0.001). No significant differences were observed in the proportion of vaginal deliveries within 24 hours (92 (92.0%) vs 94 (94.0%); p = 0.780) or cesarean deliveries (8 (8.0%) vs 6 (6.0%); p = 0.780). The groups did not differ in uterine hyperstimulation (5% vs 2%), abnormal fetal heart rate (8% vs 6%), postpartum hemorrhage (4% vs 5%), mean birth weight, or NICU admission (4% vs 0%). Conclusions: 25 µg vaginal misoprostol was associated with more effective cervical ripening and shorter time to delivery than dinoprostone gel, with fewer doses and a lower rate of oxytocin use, similar cesarean rates, and comparable maternal and neonatal safety. The use of low-dose vaginal misoprostol may represent a practical alternative to dinoprostone gel in term PROM.

## Introduction

Prelabor rupture of membranes (PROM) is defined as rupture of the fetal membranes before the onset of labor and, at term, occurs in around 8% of pregnancies. While most women with term PROM go into labor spontaneously within 24 hours, women who experience a longer interval between PROM and labor have a higher risk of developing chorioamnionitis, endometritis, and neonatal sepsis [1]. The landmark TERMPROM trial showed that induction of labor in women with term PROM decreases maternal infectious morbidity without increasing the cesarean delivery rate and is viewed more favorably by women than expectant management [2]. A later systematic review also found planned early birth to be better for women with term PROM [3].

However, recent studies indicate that the decrease in neonatal and maternal risk is most pronounced when labor is induced within 15-20 hours of rupture of membranes [4], further emphasizing the importance of an effective induction technique. If the cervix is not favorable for induction, cervical ripening must precede induction, and pharmacological ripening methods (prostaglandins) are more effective than oxytocin alone [5].

Prostaglandins are recommended as a first-line treatment for cervical ripening and induction of labor in suitable women [6]. Dinoprostone (prostaglandin E2) has long been known to be effective and is used for cervical ripening and vaginal delivery [7]. One synthetic prostaglandin E1 analog, misoprostol, is cheap, stable at room temperature, and effective for cervical ripening and induction of labor, especially in resource-limited areas [8].

Several network meta-analyses of induction agents have shown that vaginal misoprostol is one of the most effective prostaglandin induction protocols for vaginal delivery [9]. A low dose of 25 µg is now the preferred regimen because it is as effective as higher doses but has fewer uterine side effects, as uterine tachysystole is associated with higher doses [10]. The literature comparing misoprostol to dinoprostone for induction at term has reached a consensus that misoprostol is at least as effective, requires less oxytocin augmentation, and has a similar safety profile. However, most of this evidence comes from women with intact membranes, with data specific to term PROM being comparatively limited and less consistent [11]. In this context, the current study was conducted to evaluate the effectiveness and safety of 25 µg vaginal misoprostol versus dinoprostone gel for induction of labor in women with term PROM and an unfavorable cervix.

## Materials and methods

Ethical considerations, study design, and setting

Before the study began, the Institutional Ethics Committee of R.N.T. Medical College and Controller and Attached Hospitals, Udaipur, reviewed and approved the research project (approval number: RNT/STAT/IEC/2015/1485, dated December 11, 2015). Because the study retrospectively used anonymized data from hospital case records and labor-ward registers, the requirement for individual written informed consent was waived by the institutional ethics committee. This was a non-interventional observational study, as no specific intervention was designated; both medications were given as part of normal clinical practice, and prospective clinical-trial registration was not appropriate.

Participants and eligibility

Records of 200 women who met the inclusion criteria were reviewed, with 100 women in the misoprostol group (Group M) and 100 in the dinoprostone group (Group D). Inclusion criteria were singleton pregnancy, a reassuring fetal heart rate pattern at admission, cephalic presentation with an unfavorable cervix (Bishop score ≤6), PROM without spontaneous uterine contractions, and term gestational age confirmed by a reliable LMP and/or early ultrasound. Exclusion criteria included established labor at admission, previous cesarean section or other uterine scar, multiple gestation, malpresentation, clinical chorioamnionitis or overt infection at presentation, antepartum hemorrhage, non-reassuring fetal status, intrauterine fetal death, major fetal congenital malformation, or any known contraindication to prostaglandin use or vaginal delivery.

Interventions

In Group M, women received 25 µg misoprostol inserted into the posterior vaginal fornix every four hours until adequate uterine activity occurred, but no more than six doses were given. Women in Group D received 0.5 mg dinoprostone gel intravaginally as needed every six hours, up to three doses. Both regimens were similar to previously described protocols for induction in term PROM [12]. The cervix was re-examined regularly in both groups, and after completing the prostaglandin course, or if uterine activity failed to develop, oxytocin augmentation was introduced according to the institutional protocol, and intrapartum monitoring was carried out as usual. Cesarean delivery was performed for obstetric indications such as failure to progress and fetal distress.

Outcome measures

The main outcomes were induction-to-delivery interval (in hours) and the percentage of women who delivered vaginally within 24 hours after the first dose. Secondary outcomes included the Bishop score at six hours, number of induction-agent doses, induction-to-active-labor interval, need for oxytocin augmentation, and mode of delivery. Safety outcomes included uterine hyperstimulation, abnormal fetal heart rate patterns, postpartum hemorrhage, five-minute Apgar score, birth weight, and neonatal intensive care unit (NICU) admission.

Statistical analysis

We compiled the data in a spreadsheet and analyzed it using standard statistical software. For continuous variables, the data were presented as mean ± standard deviation, and an independent-samples t-test was used to compare the two groups. Categorical variables were reported as frequencies and percentages and compared using a chi-square test or Fisher's exact test, as applicable. The p-value was considered statistically significant when p < 0.05 (two-tailed).

## Results

The two study groups were comparable in baseline demographic and clinical characteristics, including maternal age, gestational age, parity, PROM duration, and baseline Bishop score (Table 1).

**Table 1:** Baseline demographic and clinical characteristics of the study groups p < 0.05 was considered statistically significant. An independent-samples t-test was used for continuous variables and a chi-square test for categorical variables. SD: standard deviation, PROM: prelabor rupture of membranes

Characteristic	Misoprostol (n = 100)	Dinoprostone (n = 100)	Test statistic	p-value
Maternal age (years), mean ± SD	25.84 ± 3.42	26.12 ± 3.58	t = −0.57	0.570
Gestational age (weeks), mean ± SD	38.42 ± 0.91	38.19 ± 0.86	t = 1.83	0.069
Parity, n (%)	-	-	χ² = 0.16	0.689
Primiparous	54 (54.0%)	51 (51.0%)	-	-
Multiparous	46 (46.0%)	49 (49.0%)	-	-
Duration of PROM (hours), mean ± SD	8.42 ± 3.16	8.67 ± 3.28	t = −0.55	0.584
Baseline Bishop score, mean ± SD	4.12 ± 1.08	4.05 ± 1.12	t = 0.45	0.652

A total of 200 women were included, with 100 in each group. The two groups had similar baseline Bishop scores, parity, PROM duration, and maternal age, suggesting a similar clinical profile at induction. The mean gestational age was slightly higher in the misoprostol group, although the difference was not statistically significant. Table 2 presents the statistical comparisons between the misoprostol and dinoprostone groups, including the corresponding test statistics and p-values.

**Table 2:** Comparison of efficacy outcomes between the study groups p < 0.05 was considered statistically significant. An independent-samples t-test was used for continuous variables, and a chi-square test was used for categorical variables. SD: standard deviation, h: hours

Outcome	Misoprostol (n = 100)	Dinoprostone (n = 100)	Test statistic	p-value
Bishop score at 6 h, mean ± SD	5.68 ± 1.31	4.78 ± 1.26	t = 4.95	<0.001
Number of doses, mean ± SD	1.62 ± 0.63	1.91 ± 0.67	t = −3.15	0.002
Induction-to-active-labor interval (h), mean ± SD	6.80 ± 1.58	8.19 ± 2.06	t = −5.35	<0.001
Induction-to-delivery interval (h), mean ± SD	11.81 ± 3.06	13.78 ± 3.99	t = −3.92	<0.001
Oxytocin augmentation, n (%)	16 (16.0%)	46 (46.0%)	χ² = 21.04	<0.001
Vaginal delivery within 24 h, n (%)	92 (92.0%)	94 (94.0%)	χ² = 0.31	0.780
Cesarean delivery, n (%)	8 (8.0%)	6 (6.0%)	χ² = 0.31	0.780

There were no significant differences in uterine hyperstimulation (5 (5.0%) in the misoprostol group vs 2 (2.0%) in the dinoprostone group), abnormal fetal heart rate patterns (8 (8.0%) vs 6 (6.0%)), or postpartum hemorrhage (4 (4.0%) vs 5 (5.0%)). Neonatal outcomes were also comparable between groups, including Apgar score <7 at five minutes (0 (0.0%) vs 1 (1.0%)) and NICU admission (4 (4.0%) vs 0 (0.0%)) (Table 3).

**Table 3:** Comparison of maternal and neonatal safety outcomes p < 0.05 was considered statistically significant. An independent-samples t-test, chi-square test, or Fisher's exact test was used as appropriate. SD: standard deviation, NICU: neonatal intensive care unit, kg: kilogram, min: minutes

Outcome	Misoprostol (n = 100)	Dinoprostone (n = 100)	Test statistic	p-value
Uterine hyperstimulation, n (%)	5 (5.0%)	2 (2.0%)	χ² = 1.33	0.445
Abnormal fetal heart rate, n (%)	8 (8.0%)	6 (6.0%)	χ² = 0.31	0.783
Postpartum hemorrhage, n (%)	4 (4.0%)	5 (5.0%)	Fisher's exact test	1.000
Apgar score <7 at 5 min, n (%)	0 (0.0%)	1 (1.0%)	Fisher's exact test	1.000
Five-minute Apgar score, mean ± SD	8.77 ± 0.81	8.66 ± 0.83	t = 0.95	0.346
Birth weight (kg), mean ± SD	2.90 ± 0.41	2.89 ± 0.36	t = 0.18	0.840
NICU admission, n (%)	4 (4.0%)	0 (0.0%)	Fisher's exact test	0.121

## Discussion

Compared with 25 µg vaginal misoprostol, 0.5 mg dinoprostone gel resulted in longer induction-to-active-labor and induction-to-delivery intervals and a greater need for oxytocin augmentation in this retrospective comparison of 200 women with term PROM and an unfavorable cervix. Importantly, the greater observed efficacy was not accompanied by significantly higher rates of uterine hyperstimulation, abnormal fetal heart rate patterns, or adverse neonatal outcomes, and cesarean delivery rates did not differ between groups.

These results agree with previous studies in the particular context of term PROM. An early trial found that vaginal misoprostol was more effective than prostaglandin E2 for preinduction cervical ripening in women with membrane rupture greater than 36 weeks and that it reduced the time to delivery and the need for oxytocin [12,13].

Randomized data have also shown the effectiveness of misoprostol as an induction agent in women with PROM, and the multicenter MisoPROM study showed that oral misoprostol is a viable alternative to oxytocin for induction in women with PROM [14].

Other trials comparing misoprostol with oxytocin for term PROM have shown similar delivery results, with the added benefit of a non-parenteral route of administration [15]. A placebo-controlled trial in women with term PROM and an unfavorable cervix also showed that misoprostol reduces time to delivery in this population and is safe [16,17].

Efficacy comparisons of misoprostol and prostaglandin E2 (PGE2) preparations have generally favored misoprostol, and an intravaginal comparison of misoprostol and oxytocin showed a shorter induction-to-delivery interval and a lower requirement for oxytocin in the misoprostol arm [18]. Our findings also align with an Indian randomized study comparing low-dose misoprostol with dinoprostone gel for cervical ripening, showing they were equally effective and safe in terms of neonatal complications and cesarean sections [19].

Physiologically, these differences reflect pharmacologic differences between the two agents. The observed differences may partly reflect pharmacologic differences between the two agents. In the present study, misoprostol was associated with a greater increase in Bishop score at six hours, shorter induction-to-active-labor and induction-to-delivery intervals, and less frequent use of oxytocin augmentation than dinoprostone gel. This efficacy benefit was observed with the low 25 µg dose, without the associated excessive uterine activity, as shown by the low and similar incidence of hyperstimulation in our cohort. From a programmatic standpoint, misoprostol is also practical in a resource-limited setting: it is affordable, can be kept at room temperature, is simple to use, and has growing evidence of efficacy in several induction indications [20].

The advantages of this study include a well-defined, clinically homogeneous population of term PROM women with an unfavorable cervix, a balanced sample of 100 women per group, and a broad range of efficacy and safety outcomes. However, there are some caveats. First, the retrospective, non-randomized design carries a risk of selection bias and confounding by indication; residual differences between groups, such as the slight imbalance in gestational age, cannot be ruled out. Second, the study took place in a single tertiary care center, which may limit how well the results apply to other health centers. Third, using routinely collected data means some outcomes, such as subtle cervical assessment or the exact timing of events, may be documented inconsistently. Finally, the sample size was large enough to detect large differences in time-based outcomes. Still, it may have been underpowered to detect clinically relevant differences in cesarean delivery rate and rare safety events. Prospective, adequately powered, randomized studies would help to confirm these observations.

Limitations

This study has several important limitations to consider when interpreting its findings. First, the study was based on clinical records from 2015; therefore, the findings reflect the obstetric practices, induction protocols, monitoring standards, and clinical decision-making prevalent during that period. Changes in clinical guidelines, induction practices, antimicrobial protocols, fetal monitoring, and availability of cervical-ripening agents over the intervening years may limit the direct applicability of these findings to contemporary clinical practice.

Second, the retrospective, record-based observational design did not involve randomization or allocation concealment. Treatment selection followed routine clinical practice and may therefore have introduced selection bias and confounding by indication. Although the available baseline characteristics, including maternal age, parity, duration of PROM, and baseline Bishop score, were comparable between the groups, unmeasured or incompletely documented clinical factors may have influenced the choice of induction agent and subsequent labor outcomes.

Third, reliance on routinely collected hospital records may have introduced information bias because the completeness and precision of documentation could not be controlled retrospectively. This may particularly affect time-dependent variables such as cervical assessment, onset of active labor, and induction-to-delivery intervals. Fourth, no a priori sample-size or formal power calculation was performed because all available eligible records were reviewed. Consequently, the study may have had limited statistical power to detect differences in uncommon outcomes, particularly rare maternal and neonatal adverse events.

Fifth, the study was conducted at a single tertiary-care teaching hospital, which may limit the generalizability of the findings to other institutions and populations. Sixth, because treatment allocation was non-randomized, interpret the observed differences as associations rather than evidence of causality or definitive superiority of one induction agent over the other. Finally, no formal cost-effectiveness analysis was performed; therefore, this study cannot support economic conclusions about the relative cost-effectiveness of misoprostol and dinoprostone.

Taken together, these limitations indicate that the findings should be interpreted in the context of the 2015 clinical setting and retrospective observational design. Contemporary, prospective, multicenter randomized controlled studies using standardized induction protocols, appropriate allocation procedures, predefined outcomes, and adequately powered safety analyses are warranted to determine whether the observed differences remain applicable to current obstetric practice.

## Conclusions

In women with term PROM and an unfavorable cervix, use of 25 µg vaginal misoprostol was associated with shorter induction-to-active-labor and induction-to-delivery intervals, fewer induction doses, and less frequent oxytocin augmentation compared with dinoprostone gel. Cesarean delivery rates and the measured maternal and neonatal safety outcomes were comparable between the groups. Given the retrospective, non-randomized design, interpret these findings as associations rather than evidence of causal superiority. Prospective randomized studies with standardized protocols and adequately powered safety outcomes are warranted to further evaluate the comparative effectiveness and safety of these induction regimens.
